# *N*-Acetyl-Aspartyl-Glutamate in Brain Health and Disease

**DOI:** 10.3390/ijms23031268

**Published:** 2022-01-23

**Authors:** Cecilie Morland, Kaja Nordengen

**Affiliations:** 1 Section for Pharmacology and Pharmaceutical Biosciences, Department of Pharmacy, The Faculty of Mathematics and Natural Sciences, University of Oslo, 1068 Oslo, Norway; 2 Department of Neurology, Oslo University Hospital, 0424 Oslo, Norway; 3 Institute of Clinical Medicine, Faculty of Medicine, University of Oslo, 0318 Oslo, Norway

**Keywords:** NAAG, retrograde neurotransmitter, glutamate carboxypeptidase II, Parkinson’s disease, Alzheimer’s disease, stroke, traumatic brain injury, pain, schizophrenia, epilepsy

## Abstract

*N*-acetyl-aspartyl-glutamate (NAAG) is the most abundant dipeptide in the brain, where it acts as a neuromodulator of glutamatergic synapses by activating presynaptic metabotropic glutamate receptor 3 (mGluR3). Recent data suggest that NAAG is selectively localized to postsynaptic dendrites in glutamatergic synapses and that it works as a retrograde neurotransmitter. NAAG is released in response to glutamate and provides the postsynaptic neuron with a feedback mechanisms to inhibit excessive glutamate signaling. A key regulator of synaptically available NAAG is rapid degradation by the extracellular enzyme glutamate carboxypeptidase II (GCPII). Increasing endogenous NAAG—for instance by inhibiting GCPII—is a promising treatment option for many brain disorders where glutamatergic excitotoxicity plays a role. The main effect of NAAG occurs through increased mGluR3 activation and thereby reduced glutamate release. In the present review, we summarize the transmitter role of NAAG and discuss the involvement of NAAG in normal brain physiology. We further present the suggested roles of NAAG in various neurological and psychiatric diseases and discuss the therapeutic potential of strategies aiming to enhance NAAG levels.

## 1. NAAG in Health

*N*-acetyl-aspartyl-glutamate (NAAG) is a *N*-acetylated dipeptide selectively localized to brain, where it is present in μM–mM concentrations [1]. This makes NAAG the most abundant dipeptide in the brain [2]. About a 10-fold difference in total tissue levels of NAAG between central nervous system (CNS) regions has been reported, with the spinal cord showing the highest levels (~23 nmol/mg protein; equivalent to roughly 2.3 mM) and pituitary showing the lowest levels (~2 nmol/mg protein; equivalent to roughly 200 µM). In the frontal cortex and the hippocampus, NAAG levels of around 3–5 nmol/mg protein were observed (equivalent to roughly 300–500 µM) [3,4]. Microdialysis data, however, do not show similar differences in basal levels of extracellular NAAG. In the dorsal hippocampus of mice, the basal extracellular levels of NAAG were found to be about 0.14 µM, increasing 15-fold (to 2.0 µM) in response to experimental traumatic brain injury (TBI) [5]. These basal data are supported by the baseline microdialysis data from the prefrontal cortex (0.10 µM) and the nucleus accumbens (0.088 µM) reported by Zuo and colleagues [6]. One should keep in mind, however, that the levels measured by microdialysis represent an average extracellular level. The levels in and adjacent to the synaptic cleft are presumably much higher.

A neuromodulatory role of NAAG in glutamatergic synapses has been extensively demonstrated. Glutamate is essential in normal neurophysiology [7] and a key component in most neurological and psychiatric diseases [8]. Hence, mechanisms to control glutamate signaling are of interest in a number of contexts and conditions. Targeting glutamate signaling directly, for instance with blockers of *N*-methyl-D-aspartate (NMDA) receptors, has been clinically challenging in most of these diseases, as the global action of NMDA blockers often induces intolerable side effects. Hence, strategies to control glutamate signaling in a more targeted manner, for instance through activity-dependent inhibition of the glutamate signal, would be preferable. The activation of the presynaptic metabotropic glutamate receptor 3 (mGluR3) represents one such strategy and can be achieved by increasing the levels of the endogenous mGluR3 agonist NAAG. Through the regulation of glutamate release, NAAG has been suggested to play a role in numerous diseases and conditions, including stroke, traumatic brain injury, epilepsy, age-related neurodegenerative diseases, schizophrenia, and pain [9].

In the present review, we summarize the transmitter role of NAAG and discuss the involvement of NAAG in normal brain physiology. We further present the suggested roles of NAAG in various neurological and psychiatric diseases and discuss the therapeutic potential of strategies aiming to enhance NAAG levels.

### 1.1. NAAG-Sensitive Receptors

**Figure 1:**
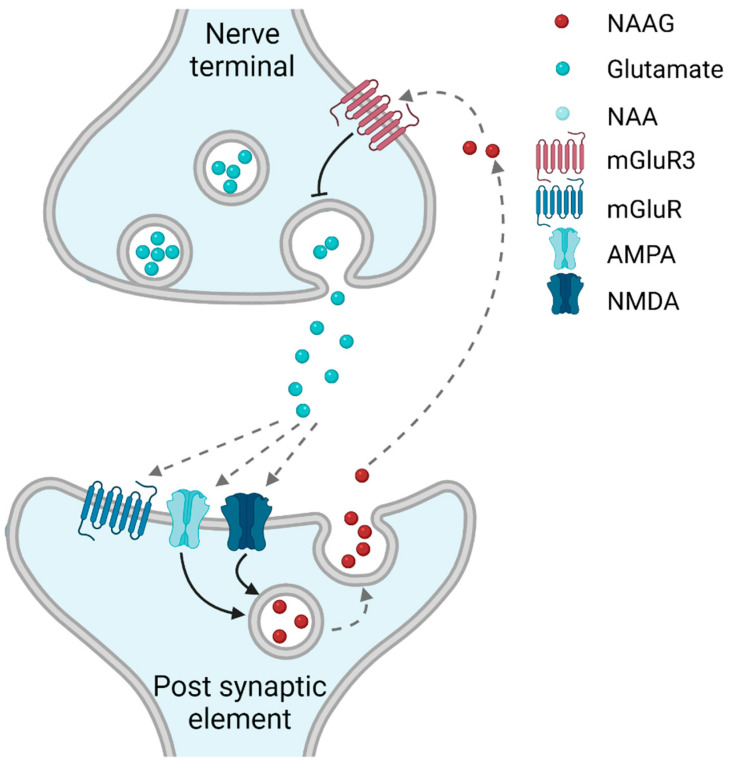
Retrograde exocytosis of N-acetyl-aspartyl-glutamate (NAAG) modulates glutamatergic neurotransmission. The postsynaptic release of NAAG is induced by the activation of postsynaptic ionotropic glutamate receptors. When released into the synaptic cleft, NAAG activates presynaptic metabotropic glutamate receptor 3 (mGluR3), reducing further glutamate release. Figure created with BioRender.

NAAG binds with high affinity to metabotropic mGluR3 (EC_50_ levels of 11–100 µM have been reported [10], depending on the assay conditions; and an IC_50_ < 5 μM was found in a displacement assay with the mGluR group II agonist LY354740). NAAG also binds with lower affinity to ionotropic NMDA receptors (EC_50_: 666 μM) [11]. Through these mechanisms, NAAG induces responses on pre-, post-, and extra-synaptic sites [12,13]. The main effect, however, appears to be through the activation of presynaptic mGluR3 [10,14], leading to the decreased release of glutamate [15]. In humans, mGluR3 is strongly expressed in the neocortex, caudate putamen, and substantia nigra, with low to moderate expression in the hippocampus, amygdala, and thalamus [16]. Contrary to what is seen in rodents, no mGluR3 appears to be present in human white matter [16]. In mice, mGluR3 has a predominantly presynaptic localization in glutamatergic synapses in the striatum and thalamus [17], but in layer III of the dorsolateral prefrontal cortex of monkeys [18] and the molecular layer of the dentate gyrus in mice [17], mGluR3 is also located in dendritic spines and astrocytic processes. Additionally, a localization on astrocytes is supported by combined in situ hybridization and immunohistochemistry, although also in this set-up, neuronal localization dominate [19]. In presynaptic terminals, mGluR3 is typically located outside of the active zone [17], see Figure 1. 

The effect of NAAG on NMDA receptors is antagonistic or agonistic [12,13], depending on the subunit composition and tissue pH [20]. As mentioned, the EC_50_ of NAAG as an NMDAR agonist is reported to be 666 μM, but an IC_50_ of 8.8 µM has been reported in a displacement assay with the NMDAR agonist CGS-19755) [12]. This dual effect on NMDA receptors opens for the possibility of neuromodulatory effects both at high and low activation levels. The release of NAAG is largely dependent on the release of glutamate (see below), but we have previously presented evidence for a basal release of NAAG even at very low glutamate signaling levels [21]. In conditions where the glutamate signal is low, NAAG has been shown to activate the synaptic, GluN2A-containing NMDARs, while inhibiting the extrasynaptic GluN2B-containing NMDARs [20]. The net result of this would be an increased signal:noise ratio. In situations of excessive glutamatergic signaling, on the other hand, GluN2B-containing NMDA receptors are important contributors to excitotoxicity. We recently reported that high glutamate and/or stimulation of ionotropic glutamate receptors enhance the release of NAAG [21]. Tissue acidification contributes to altering the effect of NAAG-binding to NMDA receptors. In these conditions, NAAG has been demonstrated to increase the activation of synaptic GluN2A-containing NMDA receptors while inhibiting extrasynaptic GluN2B-containing NMDA receptors. The physiological relevance of NAAG actions through NMDA receptors is, however, debated. Some studies find no effect of NAAG itself on these receptors, suggesting instead that the effects detected in response to administration of exogenous NAAG to cell cultures or brain slices are due to the contamination of the chemical (NAAG) with small amounts of glutamate [14].

Another suggested explanation is that the NMDA receptor actions are caused by the glutamate produced during the degradation of NAAG, while NAAG itself acts solely on mGluR3. The current belief in the field is that the neuroprotective potential of NAAG is mainly caused by the activation of presynaptic mGluR3; the effect of which is to reduce glutamate release from the nerve terminal. The activation of mGluR3 is negatively coupled to adenylyl cyclase which, in the presynaptic terminal, leads to inhibition of L-type calcium channels. The resulting reduction in voltage-dependent calcium responses inhibits exocytotic release of glutamate. As the mGluR3 agonist effect of NAAG is perceived as the most clinically important, the effects of NAAG on this receptor are often compared experimentally to well-known exogenous agonists for group II mGluR (mGluR 2 and mGluR3). A series of heterobicyclic amino acids, for instance, LY341495, LY379268, and LY354740, were developed in the lab of Darryle D. Schoepp [22]. These are highly potent (EC_50_ values in the lower nM range) but do not discriminate between mGluR2 and mGluR3. They have, however, been useful positive controls in experiments to verify the effects of NAAG through mGluR3 activation. 

### 1.2. Is NAAG a Classical (Anterograde) or an Retrograde Neurotransmitter?

To be classified as a neurotransmitter, NAAG must meet several requirements: Typically, a classical neurotransmitter is synthesized in neurons, released into the synaptic cleft by calcium- and depolarization-induced exocytosis of synaptic vesicles, and induces a response in the postsynaptic neuron by binding to plasma membrane receptors. In addition, a deactivation system must exist, securing an adequate signal:noise ratio. A retrograde neurotransmitter, on the other hand, accumulates in the postsynaptic element, is released to the synaptic cleft upon activation of postsynaptic receptors by classical transmitters, and affects the strength of the synapse by binding to presynaptic or postsynaptic plasma membrane receptors. As for the classical transmitters, the signal:noise ratio needs to be maintained through an inactivation system. 

Which of these sets of criteria are fulfilled by NAAG? In vitro studies have demonstrated that the release of NAAG from brain slices [23] and synaptosomes [24] is calcium dependent. These preparations, however, contain both pre- and post-synaptic elements [25]. Consequently, it could not be concluded as far as which compartment is responsible for the release of NAAG, based on these biochemical studies. Immunocytochemistry at the light microscopy level places NAAG in principal neurons and interneurons in the brain [26]. Based on the common belief at the time, namely that synaptic communication was strictly unidirectional, NAAG was assumed to be present in presynaptic terminals and released from this compartment. Supporting this view, a presynaptic localization of NAAG has been reported in the retina [27] and in the neuromuscular junction [28]. In the hippocampus, however, we recently demonstrated that NAAG predominantly localizes to dendritic compartments of glutamatergic synapses, both in postsynaptic spines of the Schaffer collateral synapses and in postsynaptic thorns of the mossy fiber synapses [21]. The accumulation of NAAG in synaptic-like vesicles has been suggested as sialin was found to transport NAAG when reconstructed into proteoliposomes [29]. Using postembedding immunogold labeling, we demonstrated that NAAG was concentrated in synaptic-like vesicles in the postsynaptic element and was depleted from the postsynaptic dendritic spines and thorns in response to potassium-induced depolarization and by exposure to glutamate receptor (GluR) agonists. Supporting the release of NAAG through exocytosis of postsynaptic vesicles, the postsynaptic depletion of NAAG was strictly dependent on calcium and blocked when VAMP2 (a SNARE protein reported to regulate postsynaptic exocytosis) was cleaved by botulinum toxin B. In the same study, NAAG levels in presynaptic glutamatergic or GABAergic terminals were low, and unaffected by potassium- or glutamate receptor agonist-induced depolarization [21]. These data, hence, suggest that NAAG may act as a retrograde transmitter at glutamatergic nerve terminals (Figure 1). Through retrograde NAAG transmission, the postsynaptic neuron may gain the ability to control its own inputs.

### 1.3. Synthesis and Degradation of NAAG 

The dipeptide NAAG is synthesized from *N*-acetyl-aspartate (NAA) and glutamate by NAAG synthetase (NAAGS) I and II in neurons [30,31,32]. Whether NAAGSI or II are distributed differently between subcellular compartments has not been reported. Hence, it is not known whether NAAG is synthesized selectively in presynaptic or postsynaptic elements. 

**Figure 2:**
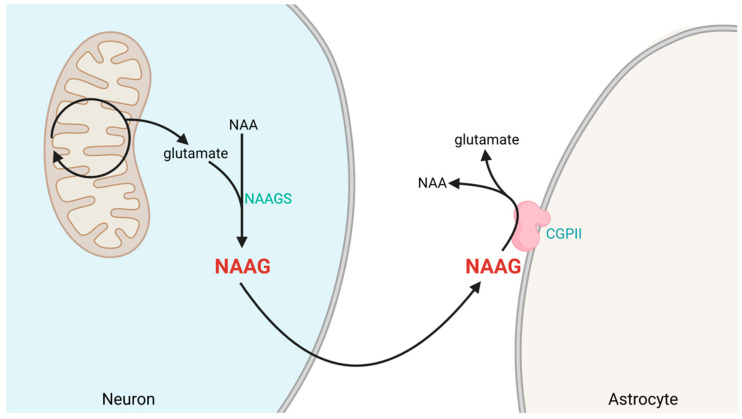
Synthesis and degradation of NAAG in the brain. NAAG is synthesized in neurons from the amino acids glutamate and N-acetyl-aspartate (NAA) via the enzyme NAAG synthetase (NAAGS). NAAG has a short extracellular half-life, as it is rapidly converted back to NAA and glutamate by membrane-bound glutamate carboxypeptidase II (GCPII) on astrocytes. Figure created with BioRender.

NAAG released to the synaptic cleft is rapidly inactivated through enzymatic degradation. Two zinc metallopeptidases, glutamate carboxypeptidase II (GCPII) and III (GCPIII), belonging to the transferrin superfamily are responsible for the cleaving of NAAG to NAA and glutamate. The higher expression levels of GCPII compared to GCPIII make GCPII the main enzyme involved in NAAG degradation. GCPII is expressed almost exclusively on the plasma membrane of glia, with the catalytic site facing extracellularly. In particular, GCPII is expressed at high levels extracellularly on astrocytes [33] and at lower levels on microglia [34]. GCPIII, on the other hand, localizes more to cerebellar and cortical neurons and less to astrocytes. The latter enzyme and its effects on NAAG neurotransmission have not been studied in detail; focus has been on the effects of GCPII. For visual illustration of NAAG metabolization, see Figure 2.

### 1.4. The Role of NAAG in Cognition

The activation of mGluR3 primarily inhibits presynaptic glutamate release [35], with positive effects on working memory [18,36]. Lower levels of NAAG, leading to the reduced stimulation of mGluR3, are associated with reduced cognitive function [34,37]. Systemic or prefrontal infusion of a GCPII inhibitor, increasing NAAG levels, improved working memory performance in young and aged rats [34] and enhances task-related neuronal firing in monkeys [18]. In addition, in humans, increased GCPII expression or gain-of-function mutations in the gene encoding GCPII (*Folh1*), which leads to decreased NAAG levels, are associated with impaired cognition [31]. Lower NAAG levels, caused by the lack of the gene encoding NAAGS I (*Rimkla* knock-out (KO) mice) or lack of the synthesis enzyme for its precursor NAA (*Nat8l* KO mice), also lead to impaired cognition [30]. On the other hand, higher NAAG levels have been reported to positively correlate with visual memory performance [31]. This positive effect of increased NAAG levels on cognition are thought to be through its effect on mGluR3 receptors, as the increased long-term memory seen when inhibiting GCPII was not seen in mGluR3 (*Grm3*) KO mice [38].

## 2. NAAG in Brain Disorders

As NAAG modulates glutamate signaling, an effect of NAAG-regulating drugs has been tested in various disease models in animals. Furthermore, endogenous NAAG levels have been studied in healthy and diseased human brains. NAAG is measured routinely in the clinic using noninvasive ^1^H-proton magnetic resonance spectroscopy (MRS), but the levels of NAAG and its precursor NAA are measured together as total NAA (tNAA). This builds on an assumption that NAAG levels mainly represent converted NAA and, hence, that NAA is the main contributor to tNAA. The findings of Menshchikov and colleagues [39] challenges this assumption. NAA and NAAG serve very different functions in brain physiology and pathophysiology, and therefore, separating these signals is highly important for avoiding the misinterpretation of the results. In this chapter, we present mechanistic in vitro work, key findings from animal models, and clinical data revealing the roles of NAAG in different neurological and psychiatric diseases. In order to study the effects of increased NAAG or prolonged action of endogenous NAAG, specific inhibitors of GCPII are valuable tools. The phosphonic acid-based GCPII inhibitor 2-(phosphonomethyl) pentanedioic acid (2-PMPA) and the thiol-based inhibitor 2-(3-Mercaptopropyl)pentanedioic acid (2-MPPA) [34] and other inhibitors (Table 1) are instrumental in demonstrating the robust therapeutic effects of GCPII inhibition and NAAG in a number of animal models of disease.

### 2.1. NAAG in Neurodegenerative Diseases

In neurodegenerative diseases, NAAG has gained increasing interest, mainly due to the selective activation of mGluR3. In the most prevalent neurodegenerative disease, Alzheimer’s disease (AD), there are significantly lower NAAG levels in the most affected brain regions, compared to in healthy individuals [45]. Increasing NAAG levels by inhibiting the NAAG peptidase GCPII was found to rescue short-term object recognition memory in an AD mouse model [38]. Mimicking the NAAG effect by adding a mGluR3 agonist proved to be neuroprotective against amyloid beta-induced neurotoxicity in mixed neuronal and astrocytic cultures [46] and pure hippocampal neurons [46]. The activation of mGluR3 has therefore been suggested as a treatment strategy in AD [47]. 

The second most prevalent neurodegenerative disease, Parkinson’s disease (PD), is characterized by the degeneration of dopaminergic neurons in the substantia nigra pars compacta (SNc), leading to motor symptoms such as resting tremor, slowness of movement, rigidity, and postural instability, in addition to nonmotor symptoms such as cognitive decline. The mechanisms underlying this degeneration remain poorly understood. The glutamatergic activity of both the corticostriatal and the subthalamic projections is dramatically increased in PD [48], and excitotoxicity has been suggested as one of several mechanisms that contribute to the cascade of events leading to dopaminergic cell death in the SNc in PD [49,50]. mGluR3 is strongly expressed throughout the basal ganglia circuitry in animals [51] and humans [52] and most prevalent in the caudate nucleus, where the mGluR3 expression is significantly lower in PD patients and PD models than in controls [52,53]. Higher glutamate release, combined with less modulatory mGluR3s in PD, forms the rationale for testing GluR3 agonists in this disease. In several PD models, improved motor activity and enhanced neuroprotection have been reported with mGluR3 agonists [54,55,56]. The effects seen with this approach suggest that NAAG and other mGluR3 agonists may represent important neuroprotective treatment options also in PD patients. 

### 2.2. NAAG in Epilepsy

Synaptically released glutamate plays a major role in the initiation and spread of epileptic activity [57,58]. The modulation of glutamatergic signaling by mGluR agonists is a possible treatment strategy [59]. Increased mGluR3 activation would lead to the inhibition of L-type calcium channels and further to diminished amounts of glutamate in the synaptic cleft. This is comparable to one of the suggested mechanisms of action for the antiepileptic drug lamotrigine, which blocks presynaptic voltage-dependent sodium channels. In rat epilepsy models, the mGluR group II agonist (2S,1′R,2′R,3′R)-2-(2,3-dicarboxycyclopropyl) glycine (DCG-IV), given prior to intraventricular injection of excitotoxic doses of kainic acid, decreased the incidence of continuous limbic motor seizures and reduced the neuronal damage [60]. Anticonvulsant effects are also shown in kindled rats, where a mGluR3 agonist was found to cause a dose-dependent increase in generalized seizure threshold [61] and to be 70-fold more potent than the anticonvulsant drug lamotrigine [61]. Another group II mGluR agonist, LY379268, reduced the electrographic course of acute status epilepticus induced by pilocarpine in mice [62]. In addition, the group II agonist (2R,4R)- 4-Aminopyrrolidine-2,4-dicarboxylate (2R, 4R-APDC) proved neuroprotective in pilocarpine-induced status epilepticus [63]. Another potent group II mGluR agonist, LY354740, inhibited neuronal excitability and epileptic activities [64] and potentiated the anticonvulsant effects of diazepam [65]. Mimicking the NAAG effect by adding mGluR3 agonist, but also increasing endogenous NAAG in the synaptic cleft through GCPII inhibition, is effectful in animal models of epilepsy [66]. Along this line, increased GCPII enzyme activity has been detected in genetically epilepsy-prone rats [67], suggesting that endogenous NAAG is rapidly degraded in this epileptic model. Further suggesting that NAAG is involved in epileptogenesis, the amount of NAAG positive neurons [68] and the expression of mGluR3 [69] have been found to be decreased in the pilocarpine model of temporal lobe epilepsy.

### 2.3. NAAG in Stroke

Ischemic stroke results in neural death through complex pathophysiological pathways, including excitotoxicity [70], oxidative stress [71,72,73,74], blood–brain barrier disruption [75], and inflammation [76]. Based on the proposed role for NAAG in preventing excitotoxicity, a neuroprotective effect in stroke seems intuitive. This hypothesis has been tested in animal models as well as cell cultures. The stage was set by two seminal papers, demonstrating that increasing NAAG with the GCPII inhibitor 2-PMPA resulted in a reduced lesion volume in response to middle cerebral artery occlusion (MCAO) in rats [77]. Furthermore, 2-PMPA was shown to efficiently protect neurons against hypoxia- or glutamate-induced toxicity [78]. The deletion of the *folh1* gene, which encodes the GCPII enzyme, is also protective in ischemia [79].

A recent study in pigs reported that the levels of NAAG in the brain tended to increase 6–12 h after a 40 min bilateral occlusion of the common carotid arteries, reaching significance at 12–24 h [32]. Simultaneously, the levels of NAAGS increased, and GCPII levels decreased. The authors propose that this self-regulation of cerebral metabolism leads to neuroprotection and enhanced neurogenesis. These results are at odds with a reported overexpression of GCPII in activated microglia in a fetal/newborn rabbit model of hypoxia/ischemia [80]. Despite the apparent variation in postischemic GCPII levels, the neuroprotective effect of increasing NAAG through either decreased degradation or increased synthesis is less controversial. In the CoCl_2_ in vitro model of ischemia, GCPII knock-down was protective against neuronal apoptosis [81]. This antiapoptotic effect could be replicated in the rat MCAO model in vivo, where a local injection of small-interfering RNA (siRNA) for GCPII resulted in a lower number of apoptotic cells in the penumbra area. Increasing NAAG levels either by the inhibition of GCPII [82] or by intraperitoneal administration of NAAG [83,84] has also been shown to reduce neuroinflammation, oxidative stress, hippocampal release of glutamate, and neurodegeneration in neonatal hypoxic-ischemic brain injury [84].

### 2.4. NAAG in Traumatic Brain Injury

Traumatic brain injury (TBI) is an acquired brain injury, resulting from external forces that damage the brain tissue. Common causes of TBI are violent hits to the head or objects penetrating through the skull and into the brain. The pathophysiology of TBI is complex [85], involving neuroinflammation [86,87], oxidative stress [88], mitochondrial dysfunction [89,90], demyelination, and other mechanisms [91]. Excitotoxicity is at the core of neural loss in response to TBI. Hence, strategies to increase NAAG concentrations in or around the affects brain area may be beneficial to counteract the excessive glutamate signaling. 

In fact, data from human patients demonstrate that NAAG levels are increased in the acute phase of TBI. These data were obtained using noninvasive MRS. Veeramuthu and colleagues [92] also reported reduced tNAA by MRS in the acute phase of TBI. Although these authors did not report NAAG levels separately, they show a larger reduction in tNAA that cannot be explained by the reduction in NAA alone. Hence, their data may support a reduction in NAAG levels ([92] and personal communication with Prof. Ramli). Theoretically, such an increase in NAAG levels in the brain would, though the activation of the mGluR3 receptor, lead to reduced excitotoxicity and hence contribute to neuroprotection. 

Direct evidence for a protective effect of NAAG in TBI have been obtained in animal models, starting with a series of papers from Bruce G. Lyeth’s lab. Using the moderate lateral fluid percussion model, Zhong and colleagues [93] demonstrated a neuroprotective effect of inhibition of GCPII in TBI. The authors treated the rats with three intraperitoneal injections of the GCPII inhibitor (S)-2-[3-[(S)-1-carboxy-3-methylbutyl]ureido]pentanedioic acid (ZJ43) at 0, 8, and 16 h post-TBI and reported a robust decrease in neuronal degradation [41]. Further supporting the role of NAAG in neuroprotection after TBI, they demonstrated that the inhibition of mGluR 2/3 by LY341495 blocked the neuroprotective effects of ZJ43. These findings were supported by Feng and coworkers, who demonstrated that a diester prodrug for ZJ43, PGI-02776, also had neuroprotective abilities in TBI [44] as well as in TBI with secondary hypoxia [94]. The same authors further demonstrated that PGI-02776 could alleviate the impairment in cognitive and motor function after TBI-hypoxia [95]. In line with this, the levels of NAAG increase in TBI, as demonstrated by microdialysis of rat hippocampus after TBI and administration of ZJ43 [5]. Simultaneously, ZJ43 prevented the rise in extracellular levels of excitatory neurotransmitters in response to TBI; the latter effect could be blocked by the coadministration of LY341495. The counteracting effect of the mGluR2/3-inhibitor and ZJ43 suggests that NAAG predominantly act through mGluR3 activation and that the reduced production of glutamate during GCPII inhibition is of less importance. GCPII KO mice have been generated and demonstrate normal neurodevelopment. These KO mice have, however, been reported to be less susceptible to oxidative stress [96], neural damage, and impairments in motor and cognitive functions in experimental TBI [97].

An alternative approach to the inhibition of GCPII is to overexpress or overactivate NAAGS. Currently, there are no known treatments that lead to increased NAAGS activity, but Li and colleagues [98] implanted neuronal stem cells that overexpressed the active NAAGS enzyme. Using this approach, they reported protection against neuroinflammation and neuronal apoptosis, followed by an improvement in post-insult motor and memory function.

### 2.5. NAAG in Pain

Sensory neurons residing in the dorsal root ganglia convey pain signals from nociceptors in the periphery to the synaptic contacts in the dorsal horn of the spinal cord. Glutamate is the primary transmitter involved in pain signaling at the spinal level, and the role of ionotropic glutamate receptors in this process is well described [99,100]. The role of mGluRs, on the other hand, is only partly understood. Pharmacological studies in animals suggest that group II mGluRs are important modulators of nociception [15], but the lack of subtype-specific antagonists to separate mGluR2 and mGluR3 action precludes the conclusion about which of these receptors are involved in mediating pain. The expression of mGluR3 in laminas II-IV has, however, been reported to increase in response to inflammatory hyperalgesia [101] while the expression of other mGluRs, including mGluR2, was unaffected. The neuronal localization of mGluR3 [101] and NAAG itself [26,102] in major sites for modulation of nociceptive transmission of the dorsal horn, along with the localization of GCPII on astrocytes in the same areas [103], opens a modulatory role of NAAG in the pain pathways. 

Interestingly, different modalities of pain involve NMDA receptors to different degrees. For instance, intrathecal administration of NMDA receptor antagonists was found to diminish allodynia evoked by carrageenan-induced inflammation of the hind paw [104,105]. The same treatment was not effective in allodynia evoked by skin incision [106] or thermal injury [107]. The idea that the maintenance of allodynia induced by carrageenan injection is mechanistically different from several other forms of hyperalgesia was strengthened by the finding that intravenous [108] or intrathecal injection of the GCPII inhibitor 2-PMPA [109,110] or intrathecal injection of the group II mGluR agonist LY379268 [111] attenuated the level of allodynia induced by carrageenan or formalin but was ineffective in allodynia induced by hypothermia or skin incisions. These studies imply that NAAG signaling has a modulatory role in mechanical-/inflammatory-induced pain. 

Further studies suggesting that NAAG signaling is involved in pain derived from the group of Joseph H. Neale. Using ZJ43 and other GCPII inhibitors, they demonstrated a role of NAAG in inflammatory pain as well as in neuropathic pain [112,113]. An indirect, long-lasting effect of GCPII inhibition by 2-PMPA has been found in pain, but the mechanisms involved have not be clarified [114]. To sum up, NAAG seems to be involved in pain, mainly through the activation of mGluR3. Different GCPII inhibitors, which increase endogenous NAAG levels, have pain-relieving effects. The selective effect of NAAG in pathways where glutamate is elevated suggests that GCPII inhibitors may represent a more targeted strategy for pain relief than a direct inhibition of the ionotropic glutamate receptors.

### 2.6. NAAG in Schizophrenia

Schizophrenia is a neurodevelopmental disorder characterized by relapsing psychotic episodes. These episodes include a set of positive symptoms: hallucinations, delusions, paranoia, and disoriented thinking, as well as negative symptoms: social withdrawal and apathy. The hyperfunction of the mesolimbic dopaminergic pathway is believed to underlie the positive symptoms, while a hypofunction of the mesocortical dopaminergic pathway is believed to form the basis of the negative symptoms. Hence, a “dopamine-hypothesis” for psychosis has evolved, and antipsychotic medications have a strong antidopaminergic component, predominantly via antagonism at dopamine D2 receptors [115]. Other neurotransmitter systems are, however, also involved in the pathophysiology of schizophrenia: for instance, a “glutamate hypothesis” has emerged [8,116] alongside the dopamine hypothesis [117]. The glutamate hypothesis builds on the evidence that the NMDA channel blockers phencycline and ketamine induce schizophrenia-like symptoms in humans. Similarly, these drugs, as well as dizocilpine (MK-801) which is used to block the NMDA receptor experimentally, induces psychosis-like behaviors in rodents [118]. Furthermore, beneficial effects in prodromal symptoms of schizophrenia have been reported for the NMDA co-agonist D-serine [119], perhaps due to its involvement in the dopamine-glutamate dialogue [120].

Several discoveries led to the suggestion that strategies to increase NAAG signaling represented novel treatment options in schizophrenia. The finding that that NAAG activates mGluR3 [14,121] combined with the demonstrations that polymorphisms in the gene encoding mGluR3 are associated with schizophrenia [122,123] gave the first clues. Furthermore, the selective mGluR2/3 agonist LY404039 was fund to attenuate the symptoms of phencyclidine-induced schizophrenia in rodents [124,125]. Supporting a role for NAAG in the pathophysiology of schizophrenia, increased GCPII and reduced mGluR3 protein levels have been described in the dorsolateral prefrontal cortex of schizophrenia patients postmortem [126], and a disruption in NAAG-mediated signaling has been suggested in the postmortem schizophrenic anterior hippocampus [127].

The effectiveness of the GCPII inhibitors ZJ43 and 2-PMPA were tested in animal models of schizophrenia. In a series of papers, Joseph H Neale’s group demonstrated that ZJ43 substantially reduced positive and negative behaviors [41] in a mGluR2/3-dependent manner and in a variety of schizophrenia models [128]. Using KO mice for mGluR2 (*Grm2*) or mGuR3 (*Grm3*), the same authors were able to ascribe the antipsychotic effects of GCPII inhibition to mGluR3 selectively [129]. Lately, NAAG has also been suggested to play a preventive role in addiction [130,131].

## 3. Translational Considerations 

As described above, neuroprotective effects of NAAG have been demonstrated in numerous neurological and psychiatric conditions in which hyperglutamatergic neurotransmission is implicated. Examples include age-related neurodegenerative diseases, epilepsy, stroke, traumatic brain injury, epilepsy, schizophrenia, and certain types of pain. For different reasons, these preclinical results have been difficult to translate into effective treatments for human diseases, and so far, drugs targeting NAAG synthesis and degradation have not reached clinical usefulness. Although highly promising when administered in vitro or injected into the brain, the classical GCPII inhibitors 2-PMPA ad 2-MPPA face a major challenge: their hydrophilic properties preclude the penetration through the blood–brain barrier. The thiol-based GCPII inhibitor 2-MPPA (GPI5693) has been tested in a double-blind, placebo-controlled, exploratory study in healthy humans [132]. Although apparently well tolerated in healthy humans, further testing was stopped as immunotoxicity was suggested in the required animal toxicity studies. These immunogenicity findings were reported to not be related to the GCPII inhibition per se but rather to the thiol-containing structure [133]. A therapeutic potential of GCPII inhibitors has also been suggested in prostate cancer and inflammatory bowel disease [134,135,136,137], where the penetration of the blood–brain barrier is not an issue [133]. The urea-based NAAG analogue, ZJ43, inhibits both GCPII and GCPIII but does not activate mGluR3 or NMDA receptors [41]. Neither ZJ43 nor its ester-prodrug PGI-02776 have been reported to have been tested in clinical trials, but both appear to pass the blood–brain barrier and induce neuroprotection in animals [44].

Barbara Slusher and colleagues recently reported to have synthesized prodrugs of non-thiol-based GCPII inhibitors with enhanced abilities to pass the blood–brain barrier [43,138]. They also explored intranasal administration [139] of GCPII inhibitors. These approaches give some promise to GCPII inhibitors reaching clinical practice in the future.

## Figures and Tables

**Table 1:** Inhibitors of glutamate carboxypeptidase II. IC_50_, half maximum inhibitory concentration; Ki, inhibition constant; and BBB, blood–brain barrier.

Inhibitor	IC50	Ki	Measuring System	Crosses BBB	References
2-PMPA: 2-(phosphonomethyl)pentanedioic acid)	-	275 pM	Synaptosomal protein preparation; detection of [3H]Glu produced from [3H]NAAG	No	Jackson et al., 1996 [40]
	4.1 nM	1.4 nM	Chinese hamster ovary (CHO) cell transfected with the cloned human GCPII; fluorescence detection of produced glutamate.		Olszewski et al., 2004 [41]
2-MPPA: 2-(3-Mercaptopropyl)pentanedioic acid	30 nM	90 nM	Purified human recombinant GCPII; detection of [3H]Glu produced from [3H]NAAG	No	Majer et al., 2003 [42]
Tetra-ODOL-prodrug for 2-PMPA	Enhanced oral bioavalability; effective after conversion to 2-PMPA		Peroral bioavailability testes in CD-1 mica and beagle dogs	Yes	Dash at el., 2019 [43]
ZJ43: (S)-2-[3-[(S)-1-carboxy-3-methylbutyl]ureido]pentanedioic acid	2.4 nM	0.8 nM (GCPIII: 23 nM)	Chinese hamster ovary (CHO) cell transfected with the cloned human GCPII or GCPIII; fluorescence detection of produced glutamate.	Yes	Olszewski et al., 2004 [41]
PGI02749: Mono-ester prodrug of ZJ43	Weak effect per se. Effective after conversion to ZJ43		Chinese hamster ovary (CHO) cell transfected with the cloned human GCPII or GCPIII; fluorescence detection of produced glutamate.	Yes	Feng et al., 2011 [44]
PGI-02776: Di-ester prodrug of ZJ43	Moderate effect per se (IC50 ~100 µM). Effective after conversion to ZJ43		Chinese hamster ovary (CHO) cell transfected with the cloned human GCPII or GCPIII; fluorescence detection of produced glutamate.	Yes	Feng et al., 2011 [44]
